# The Inhibition of Gag-Pol Expression by the Restriction Factor Shiftless Is Dispensable for the Restriction of HIV-1 Infection

**DOI:** 10.3390/v16040583

**Published:** 2024-04-10

**Authors:** Niklas Jäger, Shreya Ahana Ayyub, Frank Peske, David Liedtke, Jens Bohne, Markus Hoffmann, Marina V. Rodnina, Stefan Pöhlmann

**Affiliations:** 1 Infection Biology Unit, German Primate Center–Leibniz Institute for Primate Research, 37077 Göttingen, Germany; mhoffmann@dpz.eu; 2 Faculty of Biology and Psychology, University Göttingen, 37073 Göttingen, Germany; 3 Max Planck Institute for Multidisciplinary Sciences, 37077 Göttingen, Germany; shreya.ayyub@mpinat.mpg.de (S.A.A.); frank.peske@mpinat.mpg.de (F.P.); david.liedtke@mpinat.mpg.de (D.L.); rodnina@mpinat.mpg.de (M.V.R.); 4 Institute of Virology, Hannover Medical School, 30625 Hannover, Germany; bohne.jens@mh-hannover.de

**Keywords:** −1 programmed ribosomal frameshifting, −1PRF, Shiftless, SFL, C19orf66, HIV restriction factor, HIV-1, murine leukemia virus, stop-codon readthrough

## Abstract

The interferon-induced host cell protein Shiftless (SFL) inhibits −1 programmed ribosomal frameshifting (−1PRF) required for the expression of HIV-1 Gal-Pol and the formation of infectious HIV-1 particles. However, the specific regions in SFL required for antiviral activity and the mechanism by which SFL inhibits −1PRF remain unclear. Employing alanine scanning mutagenesis, we found that basic amino acids in the predicted zinc ribbon motif of SFL are essential for the suppression of Gag-Pol expression but dispensable for anti-HIV-1 activity. We have shown that SFL inhibits the expression of the murine leukemia virus (MLV) Gag-Pol polyprotein and the formation of infectious MLV particles, although Gag-Pol expression of MLV is independent of −1PRF but requires readthrough of a stop codon. These findings indicate that SFL might inhibit HIV-1 infection by more than one mechanism and that SFL might target programmed translational readthrough as well as −1PRF signals, both of which are regulated by mRNA secondary structure elements.

## 1. Introduction

The innate immune response is an important first line of defense against viral infection. An integral component of the innate response is the interferon (IFN) system, which senses viruses and, by producing IFN, triggers the expression of roughly 400 interferon-stimulated genes (ISG), many of which encode proteins that restrict virus infection (restriction factors) [1]. Several restriction factors have been identified that inhibit human immunodeficiency virus (HIV) infection, including APOBEC3G [2], BST2/tetherin and SAMHD1 [3], which block discrete steps of viral replication, such as reverse transcription (APOBEC3G and SAMHD1) or the release of progeny particles from infected cells (tetherin). The cellular protein Shiftless (SFL), also known as *C19orf66* [4], is a recently identified HIV-1 restriction factor that is upregulated by type I, II and III IFNs [5,6,7] and interferes with the expression of the HIV-1 Gag-Pol polyprotein, which contains the structural proteins p17, p24, p7 and p6 and the viral enzymes protease (PR), reverse transcriptase (RT) and integrase (IN) and is required for the formation of infectious viruses.

A known target of the antiviral activity of SFL is a −1 programmed ribosomal frameshifting (−1PRF) event that is essential for the expression of Gag-Pol [4]. −1PRF comprises ribosome backtracking by one nucleotide into an overlapping open reading frame (ORF), thereby producing a fusion protein encoded by upstream and downstream ORFs [8,9,10,11,12]. Many retroviruses, including Rous sarcoma virus (RSV), mouse mammary tumor virus (MMTV), human T-lymphotropic virus (HTLV), simian immunodeficiency virus (SIV) and HIV, increase the coding capacity of their genomes and regulate relative expression of viral proteins by such a strategy [4,11,12,13,14]. SFL inhibits −1PRF on HIV-1 mRNA by binding simultaneously to the translating ribosome and the downstream frameshifting stimulating element (FSE) in the viral RNA, resulting in premature termination of translation [4,15]. In contrast, its splice variant Shiftless short (SFLS) that lacks a region required for antiviral activity (RAA) also lacks the inhibitory activity, suggesting that RAA has an important functional role. The presence of RAA is crucial for SFL binding to the HIV-1 RNA, for homotypic SFL-SFL interactions and for the inhibition of −1PRF [4,15]. However, it remains unclear how this region contributes to anti-HIV activity [15,16].

**Figure 1:**
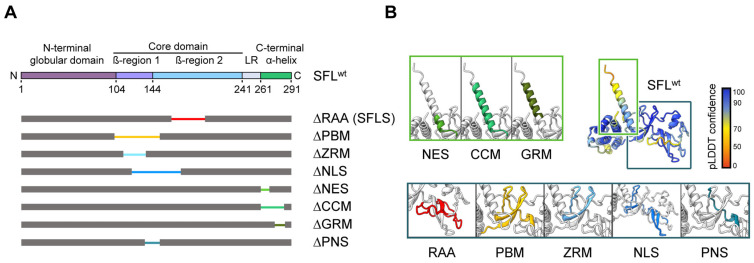
SFL structure prediction and SFL mutants analyzed. (**A**) A schematic illustration of SFL domains and regions predicted by AlphaFold2. The N-terminal globular domain (aa 1–103), the core domain (aa 104–240, including the β-region 1 (aa 104–143) and 2 (aa 144–240)), the linker region (LR; aa 241–260) and the C-terminal α-helix (aa 261–291) are indicated in SFL WT. Deleted regions are represented by narrowed colored lines: RAA, required for antiviral activity (aa 164–199; red); PBM, poly(A)-binding protein cytoplasmic 1-binding motif (aa 102–150; yellow); ZRM, zinc ribbon motif (aa 112–135; light blue); NLS, nuclear localization signal (aa 121–173; blue); NES, nuclear export signal (aa 261–269; light green); CCM, coiled-coil motif (aa 261–285; green); GRM, glutamic acid-rich motif (aa 276–286; dark green) and the overlapping region of PBM and NLS termed PNS (aa 135–150; teal blue). Delta indicates the lack of the denoted region. (**B**) SFL structure prediction by AlphaFold2. pLDDT score indicates the confidence of the structure prediction for SFL WT. Regions deleted in SFL mutants are color-coded and shown in separate boxes.

Human SFL is a 291-amino-acid(aa)-long protein with a tendency to form oligomers [15]. According to AlphaFold2 predictions [17,18], the protein contains an α-helical N-terminal globular domain (aa 1–103) followed by the core domain formed by two small β-structure elements surrounded by unstructured loops, and a long C-terminal α-helix (aa 261–291). The core domain comprises a central β-structure with an adjacent loop (aa 104–143, in the following denoted as β-region 1) and a peripheral β-structure decorated by a short α-helix (aa 144–240, denoted as β-region 2) connected to the C-terminal α-helix by a loop (linker region: LR; aa 241–260) that wraps around β-region 1 and packs against the base of the C-terminal helix and the N-terminal domain (Figure 1, Supplementary Figure S1). Deletion of the RAA (aa 164–199) as in SFLS results in a predicted rearrangement in the β-region 2 in the core domain (Supplementary Figure S1). Other AlphaFold2-predicted structure elements include a poly(A)-binding protein cytoplasmic 1-binding motif (PBM; aa 102–150, which coincides with β-region 1) containing a zinc ribbon motif (ZRM; aa 112–135) and overlapping with a putative nuclear localization signal (NLS; aa 121–173). The C-terminal α-helix comprises a coiled-coil motif (CCM; aa 261–285), which includes a putative nuclear export signal (NES; aa 261–269) and the glutamic acid-rich motif (GRM; aa 270–286) (Figure 1). The contribution of these putative motifs to SFL-mediated inhibition of −1PRF and HIV-1 infection is largely unknown.

Here, we systematically assessed the importance of the predicted SFL structural elements for its function by deleting or mutating the elements. In addition to the structural motifs identified by AlphaFold2, we deleted the region encompassing aa 135–150 shared by the predicted PBM and the NLS motif and denoted as PNS (PBM-NLS shared region) (Figure 1). We tested all mutants for their ability to oligomerize and to inhibit Gag-Pol expression and HIV-1 infection. Additionally, we examined the impact of ZRM and PNS on the binding to the ribosome and *gag-pol* mRNA. We found that the C-terminal part of the protein encompassing the NES, CCM and GRM regions is not required for homotypic interactions and the anti-HIV-1 activity of SFL, while the integrity of ZRM and PNS is important for homotypic interactions, SFL binding to viral mRNA and ribosomes, the inhibition of Gag-Pol expression and antiviral activity. Surprisingly, we found that the mutants’ ability to inhibit −1PRF and their antiviral activity were not always linked, suggesting that SFL can restrict HIV-1 infection in a −1PRF-independent fashion. Furthermore, we found that SFL inhibits murine leukemia virus (MLV), which does not rely on −1PRF for Gag-Pol expression. Therefore, we suggest that SFL can interfere with retrovirus infection in a way that does not involve the inhibition of −1PRF and for which the underlying mechanism remains to be elucidated.

## 2. Materials and Methods

### 2.1. Cell Culture

293T (human kidney cells; DSMZ no. ACC 635) and TZM-bl (human cervical cells; NIH AIDS Research and Reference Reagent Program; RRID: CVCL_B478) cells were cultivated in Dulbecco’s modified Eagle medium (PAN-Biotech; Aidenbach, Germany) supplemented with 10% fetal bovine serum (FBS; Biochrom; Berlin, Germany) and penicillin–streptomycin (PAN-Biotech; Aidenbach, Germany) at final concentrations of 100 U/mL (penicillin) and 0.1 mg/mL (streptomycin). Transfection was carried out via calcium phosphate precipitation or using Lipofectamine 2000^TM^ (Thermo Fisher Scientific, Altham, MA, USA) as described previously [15]. Cell line authentication was performed by short-tandem-repeat analysis and microscopic examination. In addition, all cell lines were tested for the absence of mycoplasma.

### 2.2. Plasmids

The sequences of SFL (C19orf66, NCBI Reference Sequence: NP_060851.2), SFLS (NCBI Reference Sequence: NP_001295206.1) and all SFL mutants (sequences available upon request) investigated in this study were inserted into a pQCXIP vector as described in [15]. All SFL variants contained either a C-terminal cMYC or HA antigenic tag. For the generation of SFL mutants, we employed overlap–extension PCR (forward and reverse primer sequences available upon request). All PCR-amplified sequences were verified using a commercial sequencing service (Microsynth SeqLab, Göttingen, Germany). The plasmids encoding MLV-Luc, MLV Gag-Pol and VSV-G were previously described [19,20]. The generation of single cycle of HIV-1 particles was performed with previously described plasmids [21,22]. HIV-1 pNL4-3 (#ARP-114) were obtained through the NIH HIV Reagent Program, Division of AIDS, NIAID, NIH, Manassas, VA, USA, from Dr. M. Martin (GenBank: AF324493).

### 2.3. Generation of HIV/MLV Single-Cycle Particles and Transduction of Target Cells

293T cells were seeded in 12-well plates at 1.5 × 10^5^ cells per well. The following day, cells were transfected via using Lipofectamine 2000^TM^ (Thermo Fisher Scientific; Waltham, MA, USA) with 1 µg each of plasmids encoding (i) an HIV- or MLV-derived vector harboring a firefly luciferase reporter gene, (ii) HIV or MLV Gag-Pol for packaging of the respective vector and (iii) the VSV-G together with plasmids encoding SFL, SFLS or SFL mutants. For the preparation of each virus produced in the presence of SFL, SFLS, or SFL mutants, 1 well of the 12-well plate was used. The empty vector served as a control. The next day, the medium of each transfected well was replaced with 1 mL of fresh medium. At day three post-transfection, the supernatants of transfected 293T cells were collected and centrifuged for 5 min at 2800× *g* to remove cell debris, diluted at a 1:1 ratio in fresh medium and used for transduction (technical quadruplicates) of 293T cells seeded in 96-well plates. At 48 h post-transduction, cells were lysed with 50 µL of cell culture reagent (Promega, Madison, WI, USA) for 30–60 min at room temperature and 45 µL of each cell lysate was transferred into a white opaque-walled 96-well plate. After adding 45 µL of FLuc substrate (PJK, Kleinbittersdorf, Germany), transduction efficiency was determined by measuring luminescence using a plate luminometer (Hidex, Turku, Finland). For further information, see [15].

### 2.4. Production of HIV-1 NL4-3 and Infection of TZM-bl Reporter Cells

293T cells were seeded in 12-well plates at 1.5 × 10^5^ cells per well and co-transfected via calcium phosphate precipitation with 1 µg of a plasmid encoding the HIV-1 NL4-3 proviral genome and plasmids expressing SFL, SFLS, or SFL mutants. The following day, the medium was replaced and 1 mL of fresh DMEM was added to the cells. After 48 h, virus-containing supernatants were collected and centrifuged for 5 min at 2800× *g* to remove cell debris. Next, TZM-bl cells seeded in a 96-well plate at 1.5 × 10^4^ cells per well were infected with equal volumes of supernatants (diluted at 1:3 in the medium) in technical triplicates. At 48 h post-infection, TZM-bl cells were lysed with PBS/0.5% Triton-X 100 containing lysis buffer for 30 min at room temperature. Next, 45 µL of cell lysate was transferred into a white opaque-walled 96-well plate and after adding 45 µL of FLuc substrate (PJK, Kleinbittersdorf, Germany), infection efficiency was determined by measuring luminescence signals using a plate luminometer (Hidex, Turku, Finland).

### 2.5. Analysis of HIV/MLV Gag-Pol Expression by SDS-PAGE and Immunoblotting

293T cells were seeded in 12-well plates at 1.5 × 10^5^ cells per well and co-transfected via calcium phosphate precipitation with 1 µg of an expression vector coding for HIV-1 or MLV Gag-Pol and vectors encoding cMYC-tagged SFL, SFLS, or SFL mutants (1 µg each), whereas an empty expression vector served as the negative control. After 72 h, the cells were lysed with 120 µL of 2 × SDS-sample buffer (0.03 M Tris-HCl, 10% glycerol, 2% SDS, 0.2% bromophenol blue and 1 mM ethylenediaminetetraacetic acid [EDTA]) containing 10% 2-mercaptoethanol for 10 min at room temperature followed by incubation at 96 °C for an additional 10 min. Proteins in whole-cell lysate (WCL) were separated via SDS-PAGE using 12.5% polyacrylamide gels and transferred onto a nitrocellulose membrane (0.2 µm pore size; GE Life Sciences, Chicago, IL, USA). Membranes were washed with PBS containing 0.1% Tween 20 (PBS-T) and were blocked in 5% milk powder dissolved in PBS-T for 1 h at room temperature. For the detection of p160 Gag-Pol and p55 Gag, an undiluted supernatant of hybridoma cells secreting a mouse anti-p24 antibody (183-HT12-5C) was used. For the detection of MLV Gag-Pol and Gag, an MLV p30 Capsid anti-rabbit antibody (originally raised by C. Stocking, Leibniz-Institute for Virology, Hamburg) was used (1:500). The expression of SFL, SFLS, and SFL mutants bearing a C-terminal cMYC antigenic tag was detected using a cMYC specific mouse antibody (MA1-980; Thermo Fisher Scientific, Waltham, MA, USA) diluted at 1:2000 in a 5% BSA/PBS-T solution. As a loading control, β-actin (ACTB) or uL3 was detected by using anti-β-actin (A2066; Sigma Aldrich, St. Louis, MO, USA) or anti-uL3 (HPA003365; Atlas antibodies, Bromma, Sweden) antibodies diluted at 1:1000 (anti-β-actin) and 1:500 (anti-uL3), respectively, in a 5% BSA/PBS-T solution. Membranes were incubated with primary antibodies overnight at 4 °C. Primary antibody binding was detected through incubation with an anti-mouse or anti-rabbit, horseradish peroxidase (HRP)-coupled secondary antibody (Dianova, Eching, Germany) at a dilution of 1:5000 for 1 h at room temperature. Visualization of bound secondary antibodies was achieved by using a chemiluminescence (ECL) solution (0.1 M Tris-HCl [pH 8.6], 250 µg/mL luminol, 1 mg/mL para-hydroxycoumaric acid and 0.3% H_2_O_2_) in combination with the ChemoCam imaging system and the ChemoStarProfessional software (INTAS, Göttingen, Germany). Signals were quantified using the software ImageJ (version 1.54d).

### 2.6. Analysis of SFL Interactions via Co-Immunoprecipitation

293T cells were seeded in 12-well plates at 1.5 × 10^5^ cells per well and co-transfected (using Lipofectamine 2000^TM^; Thermo Fisher Scientific, Waltham, MA, USA) with 1 µg of expression plasmids coding for recombinant SFL, SFLS, or SFL mutants carrying a cMYC tag together with either SFL, SFLS, or SFL mutants fused with an HA tag at the C-terminus. An empty vector served as the control. At 48 h post-transfection, the medium was removed and cells were lysed with 200 µL of radioimmunoprecipitation assay(RIPA) buffer (150 mM NaCl, 1.0% Triton X-100, 0.5% sodium deoxycholate, 0.1% SDS and 50 mM Tris, pH 8.0) containing protease inhibitor cocktail cOmpleteTM (Roche; Basel, Switzerland) for 60 min on ice. Subsequently, cell lysates were collected in 1.5 mL tubes and centrifuged for 15 min at 1800 × 10^3^× *g* at 4 °C, followed by mixing 180 µL of the supernatant with 50 µL of protein A-sepharose (Sigma; St. Louis, MO, USA; 1 g protein A-sepharose in 10 mL H_2_O) containing 1 µL of anti-HA antibody (rabbit, H6908; Sigma Aldrich, St. Louis, MO, USA). Additionally, 50 µL of the supernatant of the WCL was mixed with 50 µL of 2 × SDS-sample buffer and stored overnight at 4 °C. The protein A-sepharose–lysate mixture was incubated overnight at 4 °C in an overhead shaker. The next day, protein A-sepharose samples were centrifuged for 5 min at 1800 × 10^3^× *g* at 4 °C and the supernatant was removed. Subsequently, the samples were washed three times with 200 µL of cold RIPA lysis buffer (without protease inhibitors). Finally, 60 µL of 2 × SDS-sample buffer was added and the sampleswere further incubated for 15 min at 96 °C, before analysis via immunoblot, which was conducted as described above. For the detection of the proteins carrying an HA-epitope, an HA-specific mouse antibody (H3663; Sigma Aldrich, St. Louis, MO, USA) was used at a dilution of 1:2000 in 5% BSA/PBS-T. Analogously, proteins carrying a cMYC-epitope were detected by a cMYC-specific mouse antibody (MA1-980; Thermo Fisher Scientific, Waltham, MA, USA). As a loading control for the WCL samples, ACTB expression was detected by a β-actin-specific mouse antibody (1:2000, A5441; Sigma-Aldrich, St. Louis, MO, USA). Incubation and the imaging procedure were carried out as described above.

### 2.7. Ribosome Pelleting Assay

The ribosome pelleting assay was carried out exactly as described in [15]. Briefly, 293T cells were transfected with 2 µg of expression plasmids encoding SFL, SFLS or SFL mutants with a cMYC tag at the C-terminus. A vector expressing dsRed with a cMYC-epitope and an empty vector were used as controls. After 48 h, the cells were harvested, pelleted via centrifugation for 3 min at 2300× *g* and washed with ice-cold PBS. Cell lysis was performed using 250 µL of RNC buffer (50 mM HEPES-KOH, pH 7.4; 100 mM KCl; 5 mM MgCl_2_) supplemented with 100 µg/mL of cycloheximide and 0.1% Triton X-100. Following 15 min of incubation on ice, the lysates were centrifuged for 30 min at 13,400× *g* at 4 °C to remove cell debris. The supernatant (180 µL) was layered onto a 0.5 M sucrose cushion in RNC buffer, and the remaining lysate served as an input control. After centrifugation, the ribosomal pellet was washed, resuspended and subjected to immunoblotting for the detection of proteins of interest.

### 2.8. mRNA Binding Assay

The mRNA binding assay was carried out using the 428-nt truncated HIV-1 mRNA fragment as described in [15,23]. The mRNA was labeled with Atto488 using a modified method based on previous studies [24,25,26]. In summary, 5 nmol of mRNA was made to undergo 3′ ribose oxidation by treatment with 1 mM KIO_4_ and 0.1 M sodium acetate (pH 5.3) for 45 min on ice. The oxidation was stopped through incubation with 33 mM ethylene glycol for 10 min on ice. After two isopropanol precipitations from 0.15 M sodium acetate (pH 5.3), the mRNA was subjected to treatment with 2 mM Atto488 hydrazide (ATTO-TEC GmbH, Siegen, Germany) in 0.1 M sodium acetate (pH 5.3) and kept in the dark for 1 h at 37 °C. Following one isopropanol precipitation and one ethanol precipitation, the mRNA was dissolved in 50 µL water and quantified by measuring absorbance at 500 nm.

293T cells were plated in 12-well dishes and subjected to transfection with a recombinant SFL, SFLS or SFL mutant expression vector carrying a C-terminal MYC tag. A GFP expression vector lacking a cMYC tag served as the negative control. After 48 h of incubation, the cells underwent lysis in 450 µL of RIPA buffer and were kept on ice for 60 min. The resulting cell lysates were gathered and centrifuged for 15 min at 13,400× *g* at 4 °C, and 215 µL of the clarified lysate was combined with 25 µL of Dynabeads containing 1 µL of anti-MYC antibody and rotated for 1 h at 4 °C. Subsequently, 335 pmol of Atto488-labeled HIV-1 mRNA was introduced to the Dynabead mixture, and the samples were rotated for an additional 2 h at 4 °C. After three washes with RNC buffer using a magnetic stand (600 mM NaCl), Dynabeads were exposed to water containing 2 µg/mL of proteinase K and 0.1% SDS. Following phenol/chloroform/isoamyl alcohol extraction and ethanol precipitation, the mRNA was reconstituted and loaded onto a urea PAGE with formamide RNA loading dye [27]. The detection of Atto488-labeled mRNA was executed using the Amersham Typhoon scanner (Cytiva, Freiburg, Germany) with the Cy2 setting.

### 2.9. Statistical Analysis

Paired, two-tailed Student’s *t*-test was used to test statistical significance compared to the control (*p* > 0.05 [not significant], *p* ≤ 0.01 [*], *p* ≤ 0.005 [**] and *p* ≤ 0.001 [***]). Only values of *p* ≤ 0.05 were considered statistically significant and only statistically significant values were indicated. To test for correlation, Pearson correlation coefficients were computed. For all statistical analyses, the GraphPad Prism 7 software package was used (GraphPad Software, Boston, MA, USA).

## 3. Results

### 3.1. Deletion of the ZRM and PNS Regions of SFL Suppresses Gag-Pol Expression and Antiviral Activity

First, we sought to map the regions within SFL required for the inhibition of HIV-1 infection. For this, we generated SFL mutants lacking PBM, ZRM, NLS, NES, CCM or GRM (Figure 1) and examined their ability to restrict −1PRF-dependent expression of HIV-1 Gag-Pol and production of HIV-1 particles capable of undergoing a single cycle of infection (hereafter called single-cycle particles). The SFL splice variant SFLS, which lacks the RAA (Figure 1) and does not inhibit HIV-1 −1PRF and the production of infectious particles [4,15], was used as the control. Single-cycle HIV-1 particles were generated using an *env*, *gag* and *pol*-defective HIV-1 vector encoding firefly luciferase that was packaged using HIV-1 Gag-Pol and the vesicular stomatitis virus glycoprotein (VSV-G).

**Figure 2:**
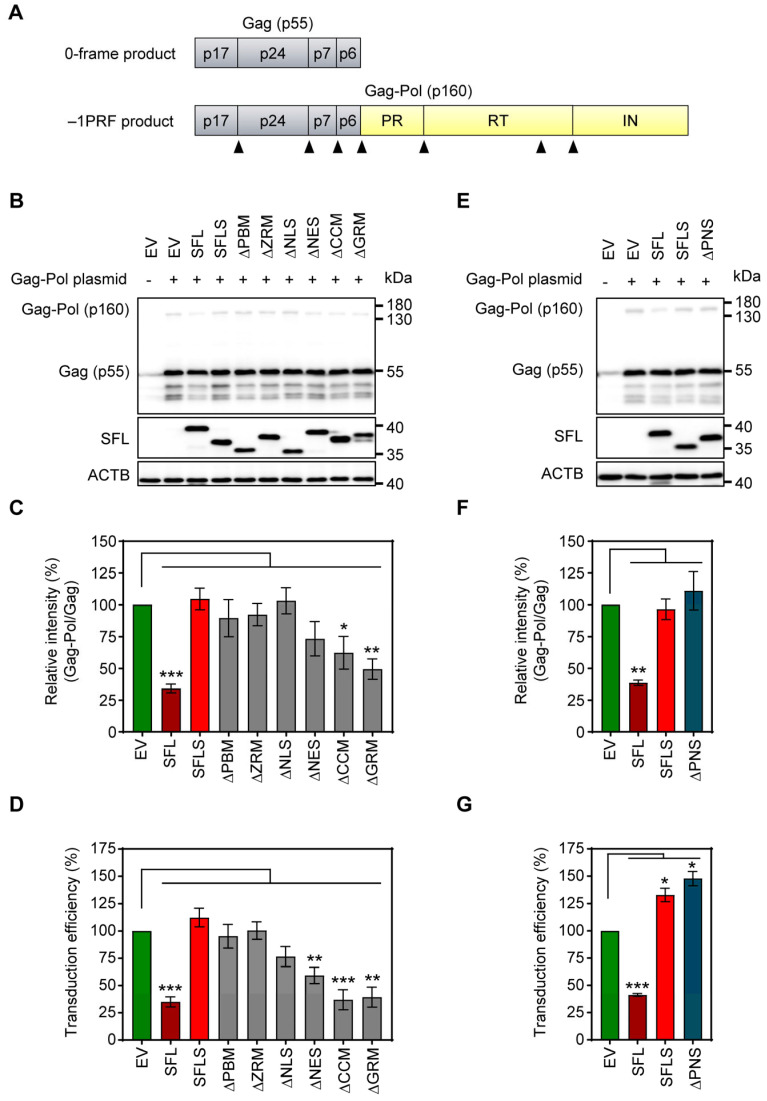
The impact of deletions on SFL expression and antiviral activity. (**A**) A schematic illustration of HIV-1 Gag (0-frame product, p55) and Gag-Pol (−1PRF product, p160) polyproteins. Gag comprises the structural proteins p17, p24, p7 and p6; Pol carries the enzymes protease (PR), reverse transcriptase (RT) and integrase (IN). Cleavage sites of the HIV protease in the Gag and Gag-Pol polyproteins are indicated by black triangles. (**B**,**E**): The inhibition of Gag-Pol expression. 293T cells were co-transfected with plasmids encoding SFL, SFLS, or SFL mutants together with a plasmid encoding the HIV-1 Gag-Pol polyprotein. Co-transfection of an empty vector served as the negative control. Cells were harvested at 48 h post-transfection and protein expression was analyzed by immunoblotting. The expression of SFL, SFLS, and SFL mutants was analyzed with an antibody against the C-terminal MYC antigenic tag. Gag-Pol (p160) and Gag (p55) expression was detected using an anti-p24 antibody. The expression of β-actin (ACTB) served as the loading control. One representative blot out of six (panel B) and one out of three (panel E) are shown, respectively. (**C**,**F**): Quantification of SFL-mediated inhibition of Gag-Pol expression. The band intensities from panels B and E were quantified and the intensity of the Gag-Pol band was normalized to that of the Gag band. Relative band intensity measured for EV transfected control cells was set as 100%. The average of six (panel B) and three (panel E) independent experiments is shown. Error bars indicate standard error of the mean (SEM). Only statistically significant values are indicated. * *p* < 0.05; ** *p* < 0.01; *** *p* < 0.001. (**D**,**G**): Transduction efficiency of single-cycle HIV-1 reporter particles produced in the presence of SFL deletion mutants. Single-cycle HIV-1 particles encoding firefly luciferase and harboring the glycoprotein of vesicular stomatitis virus (VSV-G) were produced in 293T cells co-transfected with plasmids encoding SFL, SFLS, or SFL mutants. Co-transfection of an empty vector (EV) served as the control. At 72 h post-transfection, supernatants were collected, and equal volumes were used for the transduction of 293T cells. At 72 h post-transduction, the luciferase activity in cell lysates was determined. The luciferase activity measured for supernatants from control cells (EV transfected) was set as 100%. The average of five biological replicates carried out with technical quadruplicates is shown. Error bars indicate SEM. Only statistically significant values are indicated. * *p* < 0.05; ** *p* < 0.01; *** *p* < 0.001.

To analyze SFL-dependent inhibition of Gag-Pol expression (Figure 2A), 293T cells were co-transfected with plasmids encoding Gag-Pol and SFL, SFL deletion mutants or SFLS, and cell lysates were examined for expression of SFL, Gag-Pol and Gag. The expression of SFL, SFL mutants and SFLS was readily detectable, albeit at a somewhat reduced efficiency for SFLS, ΔPBM and ΔNLS as compared to the SFL wild type (WT) (Figure 2B, Supplementary Figure S2A). The expression of SFL—but not of SFLS—reduced the ratio of Gag-Pol to Gag, as expected [4,15] (Figure 2B,C). The SFL mutants with deletions in the core domain of the protein failed to suppress Gag-Pol expression, while the mutants with C-terminal deletions, ΔNES, ΔCCM and ΔGRM, reduced the Gag-Pol to Gag ratio, albeit not to the same extent as SFL WT (Figure 2B,C).

Next, we tested whether the inhibition of Gag-Pol expression translated into antiviral activity, using an HIV-1-derived vector system. In the presence of SFL, the production of infectious particles was reduced by ~65% (Figure 2D), in accordance with published data [15]. The expression of SFLS and SFL mutants ΔPBM, ΔZRM and ΔNLS did not impair transduction efficiency (Figure 2D). In contrast, ΔNES reduced transduction efficiency by ~40%, whereas ΔCCM and ΔGRM inhibited the production of infectious particles to the same extent as SFL (Figure 2D).

The observed reduced expression of ΔPBM and ΔNLS mutants may indicate that the protein is partially misfolded and rapidly degraded in the cell. In fact, AlphaFold2 prediction suggested that ΔPBM and ΔNLS mutants lack the central β-region 1 structure (Supplementary Figure S1A). However, mutant ΔPNS, which lacks a smaller fragment comprised by 16 amino acids shared by PBM and NLS (aa 135–150) (Figure 1, Supplementary Figure S1), was robustly expressed (Figure 2E, Supplementary Figure S2B), but did not inhibit Gag-Pol expression (Figure 2E,F) and did not exhibit antiviral activity (Figure 2G). Notably, structural predictions indicate that the deletion of the PNS causes misfolding of the β-region 2, including the RAA that is missing in SFLS, which might account for the lack of antiviral activity (Figure 1, Supplementary Figure S1).

Taken together, these results suggest that deletions in the core domain inactivate SFL, whereas deletions in the C-terminal α-helix are compatible with robust SFL expression and at least partial inhibition of Gag-Pol expression and generation of infectious HIV-1 particles.

### 3.2. The Structural Integrity of the β-Region 1 Is Required for SFL Multimerization

Our previous work suggested that homotypic SFL-SFL interactions are important for the SFL anti-HIV-1 activity and that SFLS, which lacks antiviral activity, also lacks homotypic interactions [15]. To examine whether SFL deletion mutants still form multimers, we employed co-immunoprecipitation (CoIP), using SFL, SFLS, and SFL deletion mutants with C-terminal HA or MYC tags. In particular, SFL, SFLS, or SFL mutants in lysates from transfected 293T cells were immunoprecipitated with anti-HA antibody and binding partners were detected with an anti-MYC antibody via immunoblotting. Signals detected in whole-cell lysate (WCL) subjected to immunoblotting with anti-MYC or anti-HA antibodies indicate the expression levels of proteins examined for potential interactions. Further, signals in immunoprecipitates (IPs) detected with anti-HA antibodies indicate the efficiency of immunoprecipitation, while signals detected with anti-MYC antibodies indicate the efficiency of co-immunoprecipitation.

**Figure 3:**
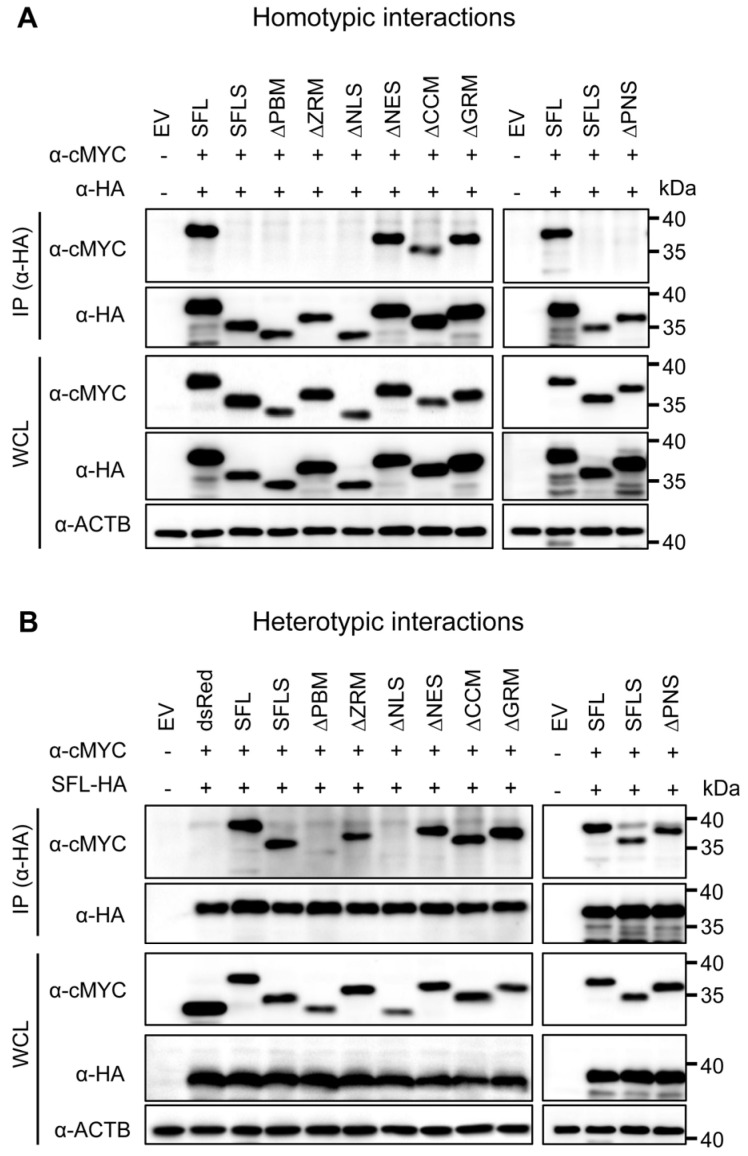
The impact of deletions on SFL self-interactions and interaction with the SFL wild type. Self-interactions (**A**) and interactions with the SFL wild type (**B**). 293T cells were co-transfected with plasmids encoding SFL, SFLS, or SFL mutants equipped with a C-terminal HA- or MYC-antigenic tag. Proteins carrying an HA tag were immunoprecipitated (IP) and detected with an antibody against the HA antigenic tag. Co-precipitated proteins were detected with an antibody against the MYC antigenic tag. The expression of the proteins in whole-cell lysate (WCL) served as the control of input proteins. The expression of β-actin (ACTB) was used as the loading control. dsRed equipped with a C-terminal MYC tag served as an additional control (panel B). Plus or minus indicates the presence (+) or absence (−) of SFL, SFLS, or SFL mutants equipped with an HA- or cMYC tag. A representative immunoblot is shown from which irrelevant lanes were excised. Similar results were obtained in two separate experiments.

We first tested the homotypic interactions using SFL as the positive control for the protein self-interaction and SFLS as the negative control, because the protein fails to self-interact [15]. cMYC-tagged SFL mutants ΔNES, ΔCCM and ΔGRM, which lack parts of the C-terminal domain and still exhibit antiviral activity, were pulled down by their HA-tagged counterparts, suggesting that they can form homo-oligomers. In contrast, mutants ΔPBM, ΔZRM and ΔNLS, which lack parts of the core domain and do not exert antiviral activity, were not pulled down (Figure 3A). However, the apparent loss of self-interaction activity of the mutants ΔPBM and ΔNLS may reflect reduced expression and/or misfolding.

Based on the observation that SFL still interacts with SFLS [15], we also tested for heterotypic interactions. SFL interacted with SFLS and SFL mutants with the exception of mutants ΔPBM and ΔNLS, which supports the notion that these mutants might be misfolded (Figure 3B). Finally, we analyzed homo- and heterotypic interactions of the ΔPNS mutant, which lacked a part of the core domain and did not have antiviral activity, although it was efficiently expressed. The ΔPNS mutant was not able to self-interact but still interacted with SFL and SFLS (Figure 3A,B). Collectively, our results indicate that the structural integrity of the core domain is important for homotypic interactions, while the C-terminal α-helix is likely not involved.

### 3.3. Inhibition of Gag-Pol Expression and HIV-1 Infectivity Are Separable Traits of SFL

**Figure 4:**
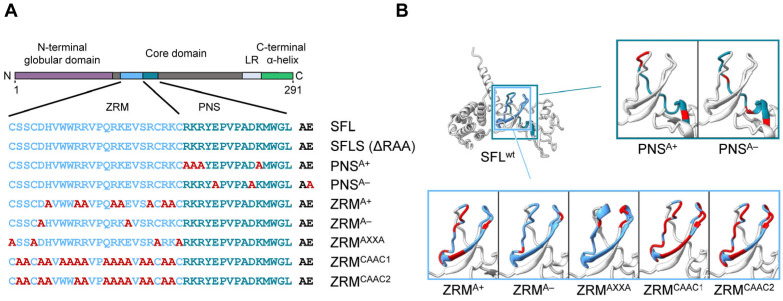
A schematic illustration and predicted protein structure of SFL alanine mutants. (**A**) SFL alanine scanning mutants. ZRM is colored in light blue and PNS in teal blue. Engineered alanine residues are shown in red. (**B**) AlphaFold2 prediction of protein structure of each SFL mutant. Amino acid residues replaced with alanine are colored in red. The PNS (teal blue), ZRM (light blue) and RAA (red) are colored in the SFL wild type.

**Figure 5:**
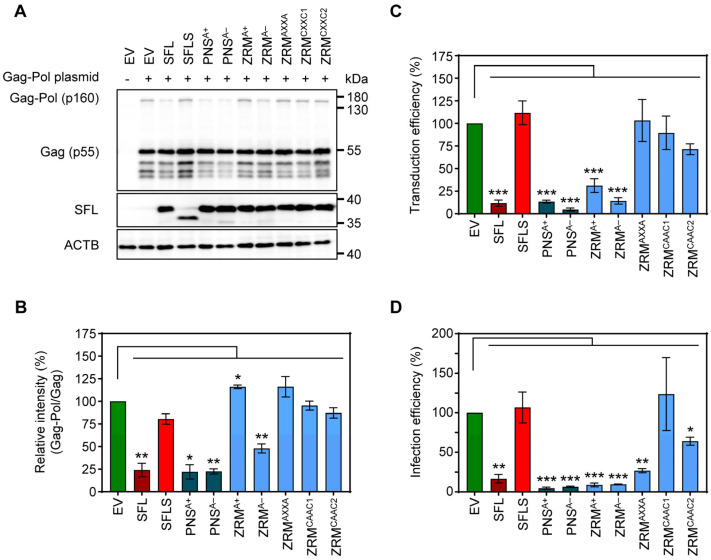
The impact of alanine mutations in ZRM and PNS on SFL antiviral activity against HIV-1. (**A**) The inhibition of Gag-Pol expression. The experiments were carried out and analyzed as described in the legend of Figure 2B,E. A representative blot out of three independent experiments is shown. (**B**) Quantification of SFL-mediated inhibition of Gag-Pol expression. The band intensities from panel A were quantified and the intensity of the Gag-Pol band was normalized to that of the Gag band. Relative band intensity measured for EV transfected control cells was set as 100%. Error bars indicate standard error of the mean (SEM). Only statistically significant values are indicated. * *p* < 0.05; ** *p* < 0.01; *** *p* < 0.001. (**C**) Transduction efficiency of single-cycle HIV-1 reporter particles produced in the presence of SFL alanine mutants. The experiments were carried out as described in the legend of Figure 2D. Transduction measured for the control particles produced in the presence of an EV was set as 100%. The average of seven biological replicates carried out with technical quadruplicates is shown; error bars indicate SEM. Only statistically significant values are indicated. * *p* < 0.05; ** *p* < 0.01; *** *p* < 0.001. (**D**) Infection efficiency of authentic HIV-1 particles produced in the presence of SFL alanine mutants. 293T cells were co-transfected with plasmids encoding SFL, SFLS, or SFL mutants and a plasmid encoding the complete HIV-1 NL4-3 genome. Co-transfection of an EV served as the control. At 72 h post-transduction, supernatants were collected and used for the infection of HIV reporter cells (TZM-bl). At 48 h post-infection, TZM-bl cells were lysed and the luciferase activity in cell lysates was determined. Luciferase activity measured upon infection with HIV-1 produced in the presence of an EV was set as 100%. The average of three biological replicates carried out with technical triplicates is shown; error bars indicate SEM. Only statistically significant values are indicated. * *p* < 0.05; ** *p* < 0.01; *** *p* < 0.001.

To further probe the effects of SFL mutants while reducing the potential effects on the overall structure, we introduced point mutations into ZRM and PNS. We have chosen to mutate basic (mutants PNS^A+^ and ZRM^A+^) and acidic (mutants PNS^A−^ and ZRM^A−^) amino acid residues to alanine (Figure 4), as basic and acidic amino acids can contribute to electrostatic protein–protein and protein–RNA interactions [28,29,30] In addition, the cysteines in the two CxxC motifs, characteristic of a zinc ribbon motif, were replaced by alanines (mutant ZRM^AXXA^). Finally, amino acid residues, which, according to the AlphaFold2 prediction, should not markedly alter the tertiary structure of SFL, were replaced by alanines (ZRM^CAAC1^ and ZRM^CAAC2^) (Figure 4). The mutations did not affect the expression efficiency compared to the SFL WT (Figure 5A, Supplementary Figure S2C). The PNS^A+^ and PNS^A−^ alanine exchange mutants were as active as the SFL WT in reducing the ratio of Gag-Pol to Gag, whereas ZRM^A−^ was slightly less active (Figure 5A,B). In contrast, mutants ZRM^A+^, ZRM^AXXA^, ZRM^CAAC1^ and ZRM^CAAC2^ did not diminish Gag-Pol expression (Figure 5A,B). Our previous study showed that SFL inhibits Gag-Pol in a dose-dependent manner [15]. However, even higher expression levels of ZRM^A+^ did not reduce Gag-Pol expression (Supplementary Figure S3A).

Mutants PNS^A+^, PNS^A−^ and ZRM^A−^ inhibited the production of infectious HIV-1 particles bearing VSV-G, in keeping with their ability to suppress Gag-Pol expression, while mutants ZRM^AXXA^, ZRM^CAAC1^ and ZRM^CAAC2^ were inactive (Figure 5B,C). Interestingly, mutant ZRM^A+^ efficiently and dose-dependently reduced the formation of infectious single-cycle HIV particles, although no inhibition of Gag-Pol expression was detected (Figure 5A–C, Supplementary Figure S3A,B).

Finally, we tested the inhibition of the authentic HIV-1 NL4-3 virus. For this purpose, 293T cells were co-transfected with a plasmid encoding the NL4-3 proviral genome and plasmids encoding SFL, SFLS, or SFL mutants. Culture supernatants were used to infect Hela cell-derived TZM-bl cells, a well-characterized HIV-1 reporter cell line [31]. The results confirmed that replacements of basic and acidic residues with alanine residues within the PNS and ZRM did not abolish SFL antiviral activity in the context of HIV-1 infection (Figure 5D, Supplementary Figure S3C). Notably, not only mutant ZRM^A+^ but also mutant ZRM^AXXA^ reduced the production of infectious HIV-1, although they did not inhibit *gag-pol* frameshifting and the latter did not interfere with the production of HIV-1 particles bearing VSV-G (Figure 5D). Finally, mutants ZRM^CAAC1^ and ZRM^CAAC2^ failed to efficiently inhibit the production of infectious single-cycle HIV-1 particles and the production of infectious HIV-1, although ZRM^CAAC2^ exerted moderate antiviral activity (Figure 5C,D). In summary, alanine substitutions of charged residues in the ZRM (ZRM^A+^ and ZRM^A−^) did not affect antiviral activity, whereas introducing multiple alanine mutations in ZRM (ZRM^CAAC1^ and ZRM^CAAC2^) largely abrogated antiviral activity, most likely due to structural alterations in the β-region 1. In addition, removing the Zn^2+^ coordination center in ZRM (ZRM^AXXA^) resulted in a loss of antiviral activity in the context of single-cycle HIV-1 particles but not in the context of authentic HIV-1.

### 3.4. Self-Interactions, Ribosome and HIV-1 RNA Binding Are Dispensable for SFL Antiviral Activity

**Figure 6:**
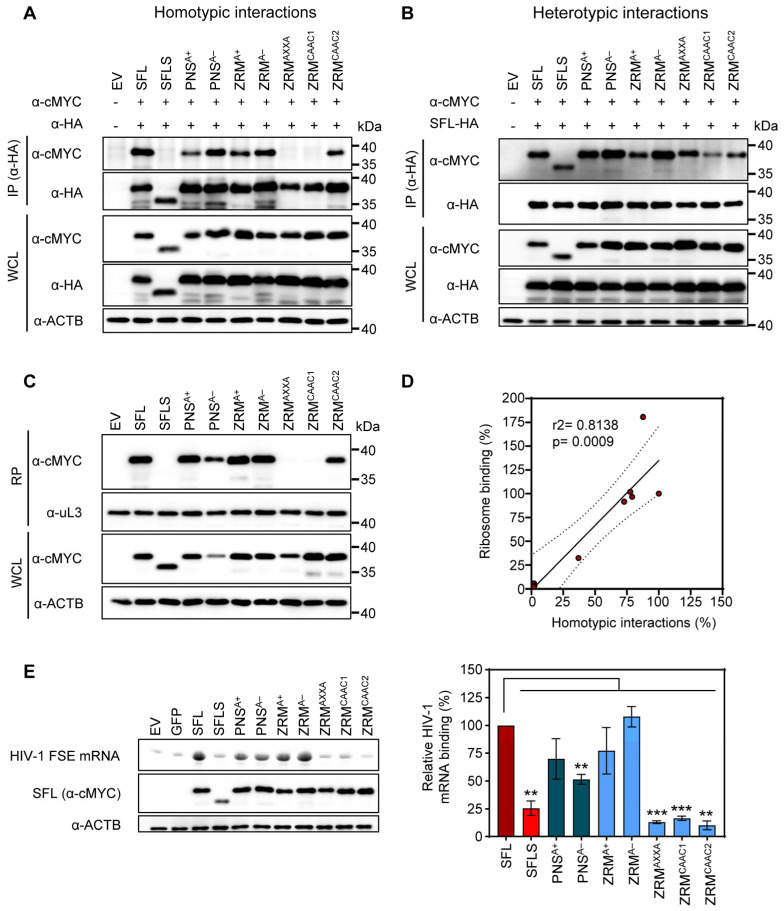
The impact of alanine mutations in ZRM and PNS on SFL self-interactions, interactions with the SFL wild type and binding to the ribosome and HIV RNA. (**A**) Homotypic interactions. (**B**) Heterotypic interactions between the SFL WT and mutants. Interactions were analyzed as described in the legend of Figure 3. A representative immunoblot is shown. Similar results were obtained in two separate experiments. (**C**) Interactions with the ribosome. SFL, SFLS, and SFL mutants’ interaction with ribosomes was detected in the ribosomal pellet (RP) using an anti-MYC antibody. WCL subjected to immunoblotting with an anti-MYC antibody indicates the expression levels of proteins examined for potential interactions. The large ribosomal subunit protein uL3 served as a loading control. A representative blot is shown. Similar results were obtained in two separate experiments. (**D**) The correlation between ribosome binding and homotypic interactions of SFL. Each dot represents one of the tested mutants, including the SFL WT and SFLS. Interactions of the SFL WT were set to 100%. (**E**) FSE RNA binding assay. Left panel, top: urea PAGE with fluorescent RNA signals, and middle and bottom panels: immunoprecipitation analysis; right panel, quantification with SFL binding to HIV mRNA was set as 100%. Error bars indicate SEM; only statistically significant values are indicated. * *p* < 0.05; ** *p* < 0.01; *** *p* < 0.001. The expression of SFL, SFLS, and SFLS mutants in WCL was confirmed via immunoblot using an anti-MYC antibody. β-Actin (ACTB) expression in WCL served as a loading control. A representative blot is shown. Similar results were obtained in two separate experiments. Fluorescence signals were measured in three separate experiments.

SFL, but not SFLS, can form multimers, associate with ribosomes and interact with HIV-1 FSE [15]. This prompted us to study whether the lack of antiviral activity of our alanine exchange mutants was due to the lack of any of these properties. CoIPs were performed as described above (see Figure 3 and the corresponding text). We observed that neither basic nor acidic residues within the ZRM or PNS were required for SFL-SFL interactions (PNS^A+^, PNS^A−^, ZRM^A+^ and ZRM^A−^) (Figure 6A). In contrast, the cMyc-tagged version of ZRM^AXXA^ and ZRM^CAAC1^ mutants were not pulled down with their HA-tagged counterparts, indicating a lack of homotypic interactions (Figure 6A). Mutant ZRM^CAAC2^ that was still able to self-interact differs from the inactive ZRM^CAAC2^ in only two tryptophan residues that remain intact in ZRM^CAAC2^ but are mutated to alanine in ZRM^CAAC1^ (Figure 4A and Figure 6A). This suggests that the two tryptophan residues are important for homotypic SFL interactions, possibility due to a contribution to the structural integrity of the ZRM. Finally, all SFL mutants were pulled down with the SFL WT, indicating that the lack of homotypic interactions, which were caused by mutations in the ZRM, are likely due to the functional inactivation of the ZRM rather than overall misfolding of the protein (Figure 6B).

Next, we investigated whether alanine mutants influenced SFL–ribosome interactions. To test ribosomal binding, cMYC-tagged SFL, SFLS, or SFL mutants were expressed in 293T cells and proteins interacting with ribosomes were detected by immunoblotting after isolation of the ribosome pellet (RP) from WCL using sucrose cushion ultracentrifugation as described previously [15]. Analysis of the RP showed that mutants capable of homotypic protein–protein interactions (PNS^A+^, PNS^A−^, ZRM^A+^, ZRM^A−^ and ZRM^CAAC2^) also interacted with the ribosome (Figure 6C), suggesting that SFL multimerization may be important for ribosomal binding and that both processes correlate with each other (Figure 6D).

Finally, we examined whether SFL mutants still bind to HIV-1 mRNA. 293T cells were transfected with plasmids encoding cMYC-tagged SFL, SFLS, or SFL mutants and cell lysates were incubated with a fluorescence-labeled mRNA containing an HIV-1 frameshifting stimulation element (FSE). SFL-RNA complexes were immunoprecipitated by Dynabeads-coupled cMYC antibodies and analyzed via urea PAGE. The signal of untagged GFP was considered background and SFL WT binding to the FSE was set to 100%. SFL but not SFLS interacted with HIV-1 RNA as expected [15] (Figure 6E). Neither basic nor acidic residues within the PNS or ZRM were essential for SFL-RNA interactions but mutating cysteine residues within the zinc ribbon motif (ZRM^AXXA^) or the replacement of most of the amino acids within the ZRM (ZRM^CAAC1^ and ZRM^CAAC2^) by alanine residues resulted in the loss of binding to the HIV-1 FSE (Figure 6E). Collectively, our results demonstrate that basic and acidic amino acid residues in the ZRM and PNS are not essential for SFL antiviral activity, protein–protein interactions, interactions with the ribosome and SFL-mRNA binding. Destruction of the zinc finger motif (ZRM^AXXA^) by mutating cysteine residues required for zinc coordination abrogates homotypic interactions, and ribosome and HIV-1 FSE binding, but not the inhibition of HIV-1 infectivity, indicating that these interactions are not essential for SFL antiviral activity.

### 3.5. SFL Inhibits MLV Gag-Pol Expression and the Formation of Single-Cycle MLV

SFL is known to inhibit HIV-1 infection by blocking −1PRF. The murine leukemia virus (MLV), which is also a member of the family *Retroviridae*, depends on the readthrough of a stop codon for Gag-Pol expression, instead of −1PRF as in HIV-1 [32,33]. However, readthrough, similarly to −1PRF, depends on a downstream secondary structure element on the mRNA [34,35]. Given the differences in the mechanism of Gag-Pol production, we wondered whether SFL inhibits the expression of MLV Gag-Pol polyprotein and the generation of infectious MLV.

**Figure 7:**
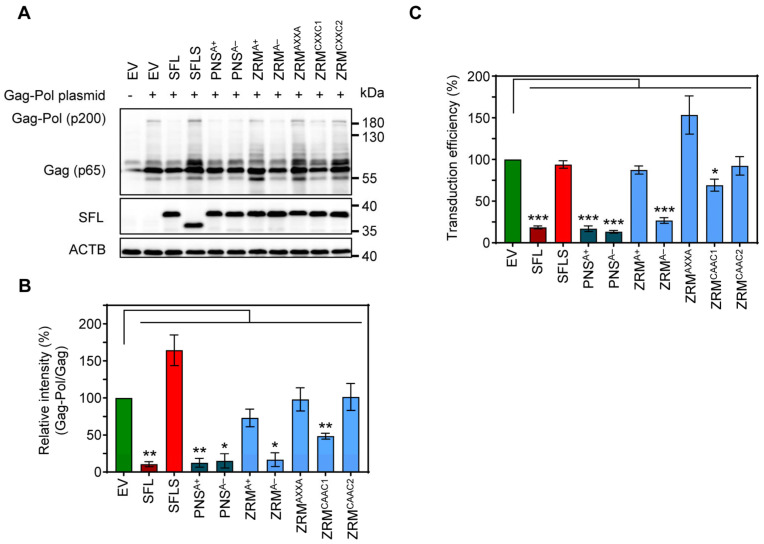
The impact of alanine mutations in ZRM and PNS on SFL expression and antiviral activity against MLV. (**A**) The inhibition of Gag-Pol expression. 293T cells were co-transfected with plasmids encoding SFL, SFLS or the indicated SFL mutants together with a plasmid expressing the friend murine leukemia virus (MLV) Gag-Pol polyprotein. Co-transfection of an EV served as the control. Cells were harvested at 48 h post-transfection and protein expression was analyzed by immunoblotting. The expression of SFL, SFLS, and SFL mutants was analyzed with an antibody against the C-terminal MYC antigenic tag. The expression of MLV Gag-Pol (p200) and Gag (p65) was analyzed using an anti-p30 antibody. The expression of β-Actin (ACTB) served as the loading control. Similar results were obtained in two separate experiments. (**B**) Quantification of SFL-mediated inhibition of Gag-Pol expression. The intensity of the Gag-Pol band was normalized to that of the Gag band. Relative band intensity measured for EV transfected control cells was set as 100%. The average of three independent experiments is shown; error bars indicate standard error of the mean (SEM). Only statistically significant values are indicated. * *p* < 0.05; ** *p* < 0.01; *** *p* < 0.001. (**C**) The transduction efficiency of single-cycle MLV particles produced in the presence of SFL alanine mutants. Single-cycle MLV particles encoding luciferase and harboring VSV-G were produced in the presence and absence of SFL, SFLS, or SFL mutants. Co-transfection of an EV served as the negative control. Supernatants were collected at 48 h post-transfection and used for the transduction of naïve 293T cells. 293T cells were lysed at 72 h after transduction and the luciferase activity in cell lysates was determined. Signals measured upon transduction with particles produced in the presence of an EV were set as 100%. The average of four biological replicates carried out with technical triplicates is shown; error bars indicate SEM. Only statistically significant values are indicated. * *p* < 0.05; ** *p* < 0.01; *** *p* < 0.001.

To determine the impact of SFL on MLV Gag-Pol expression, we co-expressed friend murine leukemia virus Gag-Pol and SFL, SFLS, or SFL mutants followed by immunoblotting with a p30 capsid-specific antibody. We found that SFL co-expression reduced the Gag-Pol-to-Gag ratio, while SFLS co-expression had no effect (Figure 7A,B). The co-expression of the SFL mutants PNS^A+^, PNS^A−^ and ZRM^A−^ had similar effects on Gag-Pol expression as the co-expression of the SFL WT, while SFL mutants ZRM^A+^, ZRM^AXXA^, ZRM^CAAC1^ and ZRM^CAAC2^ did not alter Gag-Pol expression (Figure 7A,B). In order to examine the effect of SFL on the production of infectious MLV particles, we expressed an MLV-derived vector harboring a firefly luciferase reporter gene, VSV-G and MLV Gag-Pol in the presence of SFL, SFLS, or SFL mutants, collected culture supernatants and analyzed the transduction of target cells by MLV single-cycle particles present in the supernatants. We found that the transduction efficiency of viral particles produced in the presence of SFL, PNS^A+^, PNS^A−^ or ZRM^A−^ was reduced by ~70–85%, whereas particles produced in the presence of the other SFL mutants or SFLS did not exhibit reduced infectivity (Figure 7C) despite robust expression of SFL mutants (Figure 7A, Supplementary Figure S2D). In contrast, the expression of SFL, SFLS, and SFL mutants in target cells instead of particle-producing cells had no effect on transduction efficiency, indicating no effect of SFL on viral entry (Supplementary Figure S4). Collectively, our results show that SFL is able to inhibit MLV Gag-Pol expression and the formation of infectious MLV particles.

## 4. Discussion

The present study provides evidence that the C-terminal α-helix of SFL is not required for SFL-SFL interactions and for SFL antiviral activity in the context of HIV-1 infection. In contrast, the integrity of the core domain, including both β1- and β2-regions, is essential. Furthermore, this study shows that basic amino acids in the ZRM motif are important for the inhibition of Gag-Pol expression but are largely dispensable for antiviral activity. Finally, our study demonstrates that SFL inhibits MLV Gag-Pol expression and the production of infectious MLV, which are known to be independent of −1PRF. Overall, our findings suggests that SFL can target both −1PRF and the readthrough mechanism required for retroviral Gag-Pol expression and indicate that the blockade of −1PRF and the inhibition of the formation of infectious HIV-1 are separable traits of SFL.

The present and previous studies sought to define which SFL regions are important for antiviral activity [36,37]. We found that the structural integrity of the β1-region PBM, including ZRM, NLS and PNS, is important for homotypic interactions, interference with Gag-Pol expression and antiviral activity. However, these results must be interpreted with caution, because mutations may cause global alterations in the protein’s structure that are unrelated to specific interactions with SFL ligands. This is probably the case for the ΔPBM, ΔNLS and ΔPNS mutants, as the expression of mutants ΔPBM and ΔNLS in the cell appears to be reduced compared to other mutants, and the structural predictions suggested that the overall structure of the core domain is changed (Supplementary Figure S1). In contrast, deletions of the C-terminal α-helix were at least partially compatible with homotypic interactions and antiviral activity. This argues against the notion that the C-terminal CCM of SFL is required for SFL-SFL interactions [36] and, on a more general level, suggests that SFL might tolerate alterations in the C-terminal α-helix more readily than alterations in the core domain.

Previous work suggests that SFL homotypic interactions might be required for SFL binding to the ribosome and the FSE, which in turn is needed for the inhibition of −1PRF [4,15]. However, the specific amino acid residues in SFL that are essential for protein–protein, protein–ribosome and protein–RNA interactions have not been identified. Mutants ZRM^CAAC1^ and ZRM^CAAC2^ differed in only two tryptophan residues (W119A and W120A) that were maintained in mutant ZRM^CAAC2^ but were changed to alanine in mutant ZRM^CAAC1^. Nevertheless, mutant ZRM^CAAC2^ but not mutant ZRM^CAAC1^ was capable of homotypic interactions and ribosome binding, suggesting that W119 and W120 may play an important role. One likely explanation could be that these aromatic residues are required to stabilize the folded domain [38] and that changes in folding, rather than alterations in specific side-chain interactions, are responsible for the lack of homotypic interactions of mutant ZRM^CAAC1^.

Alanine exchange of basic amino acids in ZRM^A+^ did not interfere with SFL self-interactions, SFL binding to ribosomes and binding to the HIV-1 FSE or antiviral activity. However, mutant ZRM^A+^ lost the ability to suppress the expression of Gag-Pol, indicating that the inhibition of HIV-1 infection can occur independently of the inhibition of −1PRF. In keeping with a −1PRF-independent mechanism of the inhibition of HIV-1 infection, replacement of the cysteines in the CXXC(H)-15/17-CXXC motif with alanines (ZRM^AXXA^) was compatible with the inhibition of authentic HIV-1, although this mutant neither formed multimers nor interacted with ribosomes and had lost the capability to interact with the HIV-1 −1PRF RNA and to block Gag-Pol expression. How SFL mutants inhibit HIV-1 infection without interfering with −1PRF is at present unknown but insights might come from other viruses. For instance, Kinast and colleagues found that SFL inhibited hepatitis C virus (HCV) infection by disturbing the formation of the viral replication organelle, with the cysteines in ZRM being critical for this phenotype [39]. Furthermore, SFL restricts Dengue virus (DENV) infection by interacting with RNA-binding proteins that regulate viral RNA stability and modulate the cellular translation machinery [37,40]. Thus, SFL is likely a multifunctional restriction factor able to inhibit several discrete steps in virus infection, and the full spectrum of SFL targets in HIV infection remains to be identified. This would not be unprecedented considering that, for instance, interferon-induced transmembrane proteins (IFITMs) can restrict both HIV entry and protein synthesis [41].

Why mutant ZRM^AXXA^ inhibited authentic HIV-1 but failed to block the formation of infectious single-cycle HIV particles is unclear, but one can speculate that the envelope (Env) protein may play a role. Single-cycle particles were pseudotyped with VSV-G, whereas authentic HIV-1 contained the viral Env protein. Unprocessed Gag-Pol prevents lateral movement of Env, but not of VSV-G, in the viral membrane and thereby maintains particles in a non-infectious state [42]. Whether ZRM^AXXA^ interfered with Gag-Pol processing remains to be investigated.

Numerous retroviruses rely on −1PRF, including human T cell leukemia virus type II (HTLV-2) [43], mouse mammary tumor virus (MMTV) [44], Rous sarcoma virus (RSV) and simian immunodeficiency virus (SIVmac) [45,46] and −1PRF of these viruses is inhibited by SFL [4]. However, SFL was reported to have little antiviral activity against MLV [4], which employs a readthrough instead of a −1PRF-based mechanism for Gag-Pol expression [33,35,47,48,49,50]. In contrast, we found that SFL efficiently inhibited MLV Gag-Pol expression and the production of infectious MLV particles bearing VSV-G. The reasons for the apparently differing results are unclear at present, with differences in SFL expression levels being a likely explanation. Nevertheless, our finding suggests that SFL may also inhibit programmed stop-codon readthrough in the context of MLV infection, in keeping with a previous study using cell-free systems [50]. It should be noted that both −1PRF and stop-codon readthrough depend on specific regulatory secondary structure elements, e.g., pseudoknot or stem loop structures in the viral RNA, and one can speculate that SFL targets these structures.

Collectively, our data suggest an important role of the core domain of SFL in various functions: self-interactions, association with the ribosome, binding to frameshifting RNA and inhibiting −1PRF. Additionally, we show that SFL may also target programmed readthrough, alongside−1PRF. It is tempting to speculate that SFL might bind to similar RNA elements involved in both −1PRF and programmed readthrough, yet the exact structural characteristics of these elements for SFL binding remain to be explored. Finally, our study suggests that SFL can interfere with HIV-1 infection without inhibiting −1PRF. Recent work by the Dinman group has shown that SFL overexpression leads to a reduction in mRNA abundance of both reporter and endogenous cellular mRNAs [51]. It is conceivable that the various SFL mutants that we have analyzed could impact *gag-pol* mRNA levels in a different way, possibly due to variable extents of mRNA binding or recruitment of mRNA surveillance proteins. However, it remains to be elucidated whether SFL alters viral and cellular mRNA levels in the context of retroviral infection. Understanding the complex interplay of SFL’s various functions and how they contribute to its overall anti-HIV activity is an area that still needs further exploration.

Our study has limitations. The effect of the SFL WT and mutants was examined upon directed, rather than endogenous expression, and the cellular localization of the SFL WT and mutants remains to be determined. Furthermore, a packaging plasmid derived from the HIV-1 NL4-3 genome and not authentic HIV-1 NL4-3 was employed to study the impact of SFL and SFL mutants on Gag-Pol levels, and formally, confirmation with authentic HIV-1 NL4-3 is needed. Finally, the impact of the SFL WT and mutants on the expression of viral enzymes should be determined in future studies.

## Data Availability

The data presented in this study are available on request from the corresponding author.

## Supplementary Materials

The following supporting information can be downloaded at: https://www.mdpi.com/article/10.3390/v16040583/s1, Figure S1: Structure prediction of SFL and SFL deletion mutants by AlphaFold2; Figure S2: Expression of SFL, SFLS and SFL mutants; Figure S3: Dose-dependent antiviral activity of SFL and ZRM^A+^; Figure S4: SFL expression in target cells does not interfere with transduction by single cycle HIV and MLV particles.
