# Supplementary material for: The Inhibition of Gag-Pol Expression by the Restriction Factor Shiftless Is Dispensable for the Restriction of HIV-1 Infection
Source: Viruses. 2024 Apr 10;16(4):583. doi: 10.3390/v16040583 (PMC11055011; doi:10.3390/v16040583)
Supplement: Supplementary file 1 [file viruses-16-00583-s001.zip › viruses-2920497-supplementary.pdf]

viruses

MDPI

Article

The Inhibition of Gag-Pol Expression by the Restriction Factor Shiftless Is Dispensable for the Restriction of HIV-1 Infection

Niklas Jäger [1,2,†,*], Shreya Ayyub [3,†], Frank Peske [3], David Liedtke [3], Jens Bohne [4], Markus Hoffmann [1,2], Marina V. Rodnina [3] and Stefan Pöhlmann [1,2,*]

1 Infection Biology Unit, German Primate Center – Leibniz Institute for Primate Research, 37077 Göttingen, Germany
2 Faculty of Biology and Psychology, University Göttingen, 37077 Göttingen, Germany
3 Max Planck Institute for Multidisciplinary Sciences, 37077 Göttingen, Germany
4 Institute of Virology, Hannover Medical School, 30625 Hannover, Germany
* Correspondence: njaeger@dpz.eu (N.J.), spoehlmann@dpz.eu (S.P.)
†These authors contributed equally to this work.

Supplemental Figures

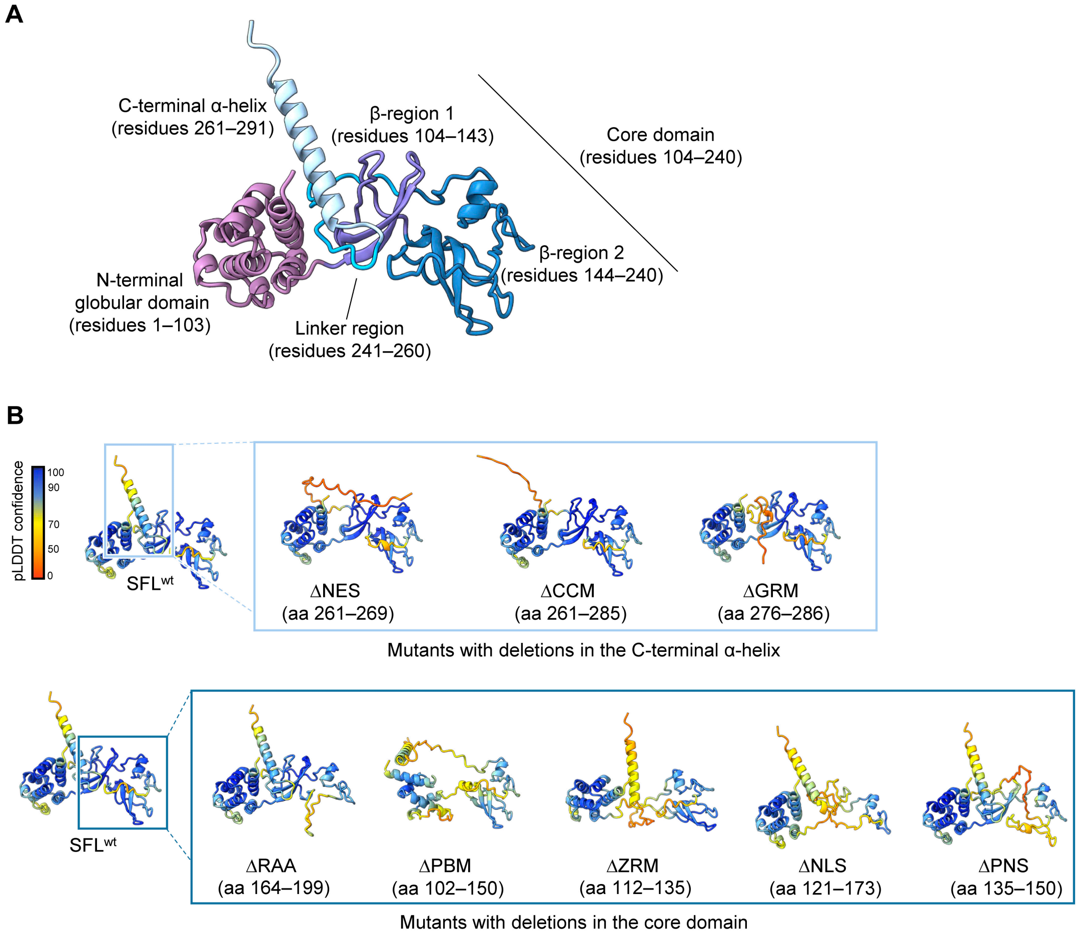

Supplemental Figure S1. Structure prediction of SFL and SFL deletion mutants by AlphaFold2. (A) Structure of SFL WT. Predicted domains and structure elements are indicated: N-terminal globular domain (aa 1-103), the core domain (aa104-240), including β-region-1 (aa 104-143) and β-region-2 (aa 144-240), the linker region (aa 241-260) and a C-terminal α-helix (aa 261-291). (B) Structures of SFL, SFLS (ΔRAA) and SFL deletion mutants. Mutants with deletions in the C-terminal α-helix (light blue) and the core domain (dark blue) are shown in separate boxes. Amino acids deleted in each mutant are indicated in brackets. pLDDT score indicates confidence of predicted structures.

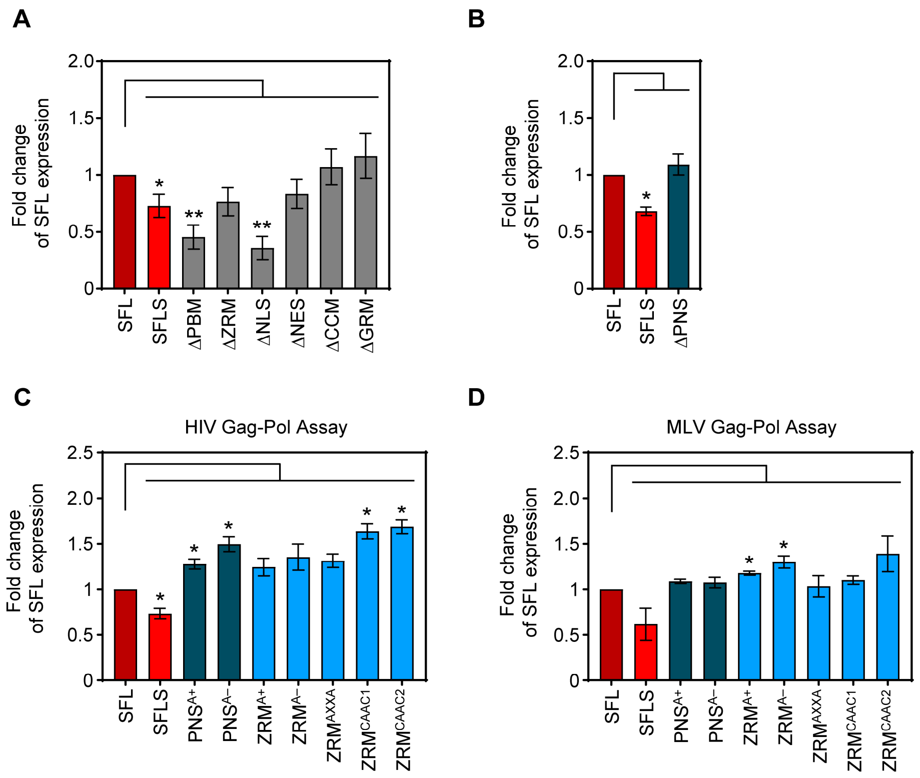

Supplemental Figure S2. Expression of SFL, SFLS and SFL mutants. Expression of SFL mutants. The band intensities from (A) Figure 2B, (B) Figure 2E, (C) Figure 5A and (D) Figure 7A were quantified and the intensities of cMYC-tagged SFL bands were normalized to that of ACTB. Relative band intensity measured for SFL WT was set as 1. The average of six (panel A) or three (panel B-D) independent experiments is shown; error bars indicate SEM; only statistically significant values are indicated; $^{*}p < 0.05$; $^{**}p < 0.01$.

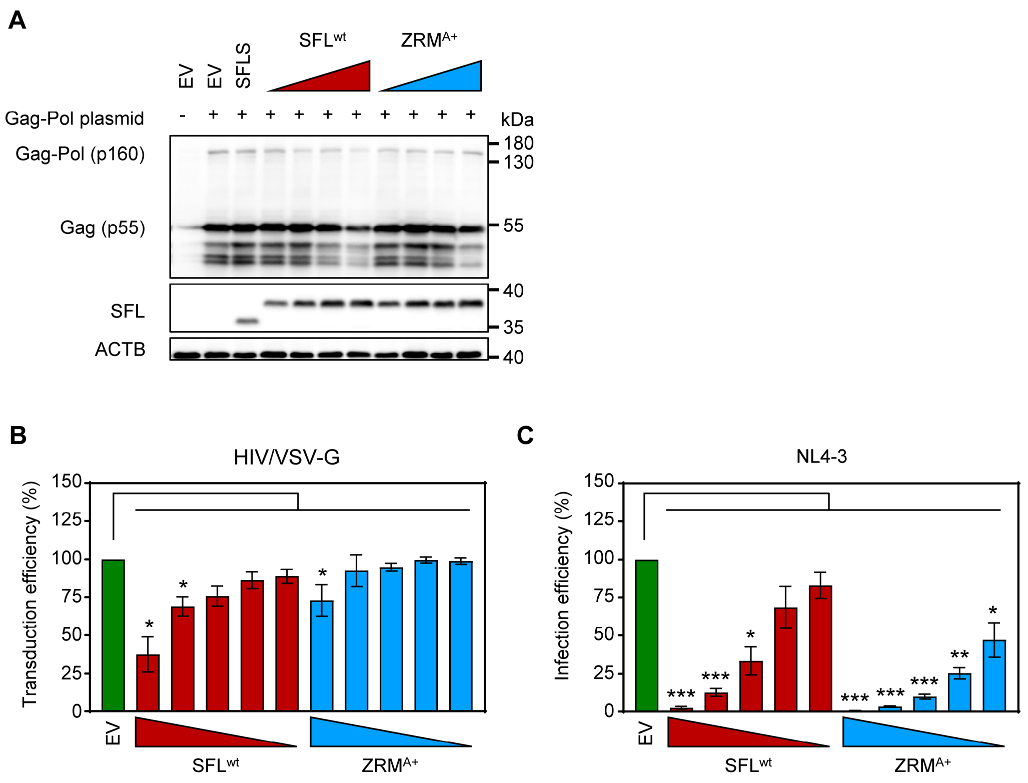

Supplemental Figure S3. Dose-dependent antiviral activity of SFL and ZRM^{A+}. (A) Inhibition of Gag-Pol expression. 293T cells were co-transfected with increasing amounts (0.5 µg, 1 µg, 2 µg and 4 µg) of plasmids encoding SFL or mutant ZRM^{A+} and a plasmid encoding the HIV-1 Gag-Pol polyprotein. Co-transfection of EV served as a control. Cells were harvested at 48 h post-transfection and protein expression was analyzed by immunoblot. Expression of SFL, SFLS, and SFL mutants was analyzed with an antibody against the C-terminal MYC antigenic tag. Gag-Pol (p160) and Gag (p55) expression was detected using an anti-p24 antibody. Expression of β-Actin (ACTB) served as loading control. A representative blot is shown. Similar results were obtained in two separate experiments. (B) Transduction efficiency of single cycle HIV-1 reporter particles produced in the presence of SFL WT or ZRM^{A+}. Particles encoding luciferase and harboring VSV-G were produced in the presence of increasing amounts of SFL WT or ZRM^{A+}. For this, 293T cells seeded in 12 well plates were co-transfected with plasmids for production of single cycle HIV-1 particles and 1 µg, 0.5 µg, 0.25 µg, 0.125 µg, 0.0625 µg of plasmid encoding SFL WT or ZRM^{A+}. Each well was transfected with 1 µg of plasmid in total replenished with EV where needed. 1 µg of EV served as a control. At 72 h post-transfection, supernatants were collected, and equal volumes were used for transduction of 293T cells. At 72 h post-transduction, the luciferase activity in cell lysates was measured. Transduction measured for control particles produced in the presence of the EV was set as 100%. The average of three biological replicates carried out with technical quadruplicates is shown, error bars indicate SEM; only statistically significant values are indicated; *$p < 0.05$; **$p < 0.01$; ***$p < 0.001$. (C) Infection efficiency of authentic HIV-1 produced in the presence of SFL WT or ZRM^{A+}. 293T cells were co-transfected with plasmids encoding SFL or ZRMA+ as described in panel B together with a plasmid encoding the HIV-1 NL4-3 proviral genome. At 72 h post-transfection supernatants were collected and HIV-1 reporter cells (TZM-bl) were infected with equal volumes of the supernatants. At 48 h post-infection, TZM-bl cells were lysed and the luciferase activity in the cell lysates determined. The average of three biological replicates carried out with technical triplicates is shown, error bars indicate SEM; only statistically significant values are indicated; *$p < 0.05$; **$p < 0.01$; ***$p < 0.001$.

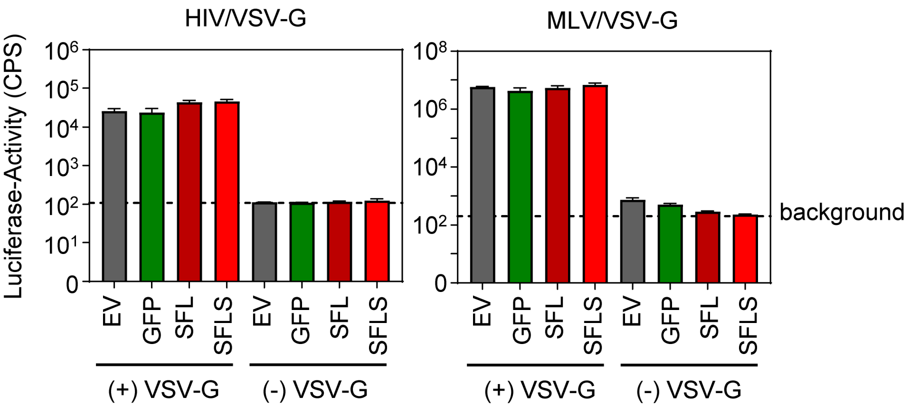

Supplemental Figure S4. SFL expression in target cells does not interfere with transduction by single cycle HIV and MLV particles. Equal volumes of culture supernatants containing single cycle HIV or MLV reporter particles were used for transduction of 293T cells previously transfected with empty vector or plasmids encoding GFP, SFL or SFLS. Particles lacking VSV-G ((-) VSV-G) served as negative controls. Cell entry was measured at 48 h post-transduction by quantifying luciferase activity (CPS) in cell lysates. The results of a single representative experiment carried out with technical triplicates are shown, error bars indicate standard deviation (SD). Similar results were obtained in three separate experiments. CPS: counts per second.
